# Fast Bootstrapping and Permutation Testing for Assessing Reproducibility and Interpretability of Multivariate fMRI Decoding Models

**DOI:** 10.1371/journal.pone.0079271

**Published:** 2013-11-14

**Authors:** Bryan R. Conroy, Jennifer M. Walz, Paul Sajda

**Affiliations:** Department of Biomedical Engineering, Columbia University, New York, New York, United States of America; Beijing Normal University, China

## Abstract

Multivariate decoding models are increasingly being applied to functional magnetic imaging (fMRI) data to interpret the distributed neural activity in the human brain. These models are typically formulated to optimize an objective function that maximizes decoding accuracy. For decoding models trained on full-brain data, this can result in multiple models that yield the same classification accuracy, though some may be more reproducible than others—i.e. small changes to the training set may result in very different voxels being selected. This issue of reproducibility can be partially controlled by regularizing the decoding model. Regularization, along with the cross-validation used to estimate decoding accuracy, typically requires retraining many (often on the order of thousands) of related decoding models. In this paper we describe an approach that uses a combination of bootstrapping and permutation testing to construct both a measure of cross-validated prediction accuracy and model reproducibility of the learned brain maps. This requires re-training our classification method on many re-sampled versions of the fMRI data. Given the size of fMRI datasets, this is normally a time-consuming process. Our approach leverages an algorithm called fast simultaneous training of generalized linear models (FaSTGLZ) to create a family of classifiers in the space of accuracy vs. reproducibility. The convex hull of this family of classifiers can be used to identify a subset of Pareto optimal classifiers, with a single-optimal classifier selectable based on the relative cost of accuracy vs. reproducibility. We demonstrate our approach using full-brain analysis of elastic-net classifiers trained to discriminate stimulus type in an auditory and visual oddball event-related fMRI design. Our approach and results argue for a computational approach to fMRI decoding models in which the value of the interpretation of the decoding model ultimately depends upon optimizing a joint space of accuracy and reproducibility.

## Introduction

Multivariate pattern analysis (MVPA) is becoming a standard tool for aggregating cortical activity across brain regions to predict various markers of cognitive state related to a task or stimulus condition [1]–[3]. In contrast to standard univariate statistical tests based on the General Linear Model (GLM) [4], MVPA uses machine learning techniques to extract task-relevant information from spatially-distributed patterns of activity [1], [5]–[14]. As a result, it has the additional benefit of being able to exploit interactions between voxels.

Oftentimes, a related goal of MVPA is to make inferences about the workings of the brain and its underlying cognitive processes. When the machine learning method produces its discriminating component by taking linear combinations of voxels, questions of inference center around interpreting the weights assigned to voxels, which is often called a “brain map.” For this reason, a wave of attention has recently been focused on developing models that are both parsimonious and interpretable. Thus, model prediction accuracy is not the only goal of the MVPA: the spatial patterns themselves are just as important.

A wide array of MVPA methods has been proposed for application to fMRI data [1], [5]–[14]. Though they differ in the assumptions made about the size and location of the spatial pattern of activity, as well as its relationship to the brain state of interest, all must grapple with the high dimensionality of the fMRI data relative to the number of trials acquired throughout the experiment. Without properly addressing this discrepancy, the learning algorithm will tend to overfit to the training data and lack generalization power. To overcome this obstacle, sometimes it is possible to identify a pre-defined anatomical region-of-interest (ROI), which greatly reduces the dimensionality of the feature space [15]. Other methods average signals across multiple ROI's or utilize some classical form of dimensionality reduction as a first step (e.g., PCA or ICA) [16]. Another option is searchlight analysis [9], which learns many spatially-localized classifiers as a “searchlight” is swept across the brain. This analysis overcomes the overfitting problem since each classifier is learned from a low-dimensional subset of the brain, but the problem lies in how to properly statistically assess the thousands of classifiers learned across the brain. By design, this method is also unable to capture interactions between spatially remote regions of the brain.

This paper focuses instead on interpreting brain maps derived from full-brain sparse regression models. In this case, feature selection and dimensionality reduction are not specified a-priori but must be learned, and are thus wrapped into the cross-validation stage of the machine learning procedure. This is usually accomplished in one of two ways. Feature selection techniques [7], [17] first perform a univariate selection strategy to identify voxels that are strongly predictive of the brain state of interest. Once the features are identified, a classifier is learned on this reduced data space. Alternatively, a number of groups have applied sparse regression models to full-brain fMRI analysis [13], [14], which allows for feature selection and classification to be performed simultaneously. This is achieved by an objective function that trades off model fit with model complexity. Here, complexity is measured by a regularization term that penalizes a combination of the length (ℓ_1_-norm) and squared energy (ℓ_2_-norm) of the regression weights [18], [19]. This penalty, called the elastic net, is known to encourage sparse solutions, so that the final predictor is derived from only a small subset of the voxels. Thus, feature selection is performed during the classification procedure. It also leads to a convex optimization problem, which greatly simplifies the optimization procedure. We restrict our attention to the latter method since it is more flexible, but we note that our approach is equally valid for the two-stage feature selection and classification procedure.

We focus on such full-brain classifiers for two reasons. First, they are data-driven and make few assumptions about the location of the brain signal of interest. This allows them to, with minimal prior knowledge, be applied to a wide array of problems and datasets. Second, interpreting brain maps from full-brain analyses is still a challenging problem that lacks a systematic tool for evaluation and interpretation.

Our approach uses a combination of bootstrapping and permutation testing to provide both a measure of cross-validated prediction accuracy and model reproducibility of the learned brain maps. This requires re-training our classification method on many re-sampled versions of the fMRI data. Given the size of fMRI datasets, this is normally a time-consuming process. We, however, make use of our recently proposed FaSTGLZ algorithm [20], [21], which was specifically designed to train many related sparse classifiers on a single dataset simultaneously. This makes our approach computationally efficient and feasible.

In conjunction with this approach, we also provide a mechanism to better visualize classification results in two-dimensions: prediction accuracy vs. model reproducibility. This is useful not only as a diagnostic tool to better understand the trade-off between these two possibly competing goals, but it also serves as a means to better inform the model selection stage of analysis. As with most discriminative methods, full-brain classification models contain regularization parameters that must be tuned [22]. The most common method is cross-validation, in which models are compared based on their predictive power. Given the present discussion, there are obvious limitations in this approach: predictive accuracy addresses how much information is encoded in the brain, but it does not speak to how reproducible and robust the derived spatial patterns are. We consider model selection as a multi-objective optimization problem and provide a principled method to properly take into account both prediction accuracy and model reproducibility. In applying this method to real experimental fMRI datasets, we show empirically that sacrificing a small reduction in cross-validated prediction accuracy generally results in a large and significant improvement in model reproducibility. This is particularly important when making inferences about activated brain regions that are common to or differ across groups. Furthermore, MVPA provides sufficient sensitivity to identify individual differences within a group, but interpretation of results is robust only when they are reproducible.

## Materials and Methods

The data used in this paper are from a previous simultaneous EEG-fMRI experimental study [23]. Only the fMRI data are used in this paper. Details on the behavioral paradigm and data preprocessing are reproduced here for completeness.

### Ethics Statement

This study was approved by the Columbia University Institutional Review Board and all subjects gave written informed consent in accordance with the guidelines of the Columbia University Institutional Review Board.

### Behavioral Paradigm

Fourteen subjects (5 female, mean 27.4 years, range 20–40) participated in three runs each of auditory and visual oddball paradigms. For each oddball detection task, 375 (125 per run) total stimuli were presented for 200 ms each with a 2–3 s uniformly distributed variable inter-trial interval (ITI) and probability of target 0.2. The first two stimuli of each run were constrained to be standards. For the auditory oddball task, the standard stimulus was a 390 Hz pure tone, and the target was a broadband “laser-gun” sound. These were selected based on troughs in the frequency spectrum of the scanner noise, and to match visual discriminator performance of the EEG data. For the visual task, the target and standard stimuli were, respectively, a large red circle and a small green circle on isoluminant gray backgrounds (3.45 and 1.15 visual angles). Subjects were asked to respond to target stimuli only, using a button press with the right index finger on an MR-compatible button response pad. Stimuli were presented to subjects using E-Prime software (PST, Pittsburgh, PA) and a VisuaStim Digital System (Resonance Technology, Northridge, CA), comprised of headphones and 600×800 goggle display.

### fMRI Data Acquisition and Preprocessing

A 3T Philips Achieva MRI scanner (Philips Medical Systems, Bothell, WA) was used to collect functional echo-planar image (EPI) data with 3 mm in-plane resolution and 4 mm slice thickness. We covered the entire cortex by obtaining 32 slices of 64×64 voxels using a 2000 ms repetition time (TR) and 25 ms echo time (TE). We also acquired a single-volume high resolution (2×2×2 mm) EPI image and a 1×1×1 mm spoiled gradient recalled (SPGR) image for each subject for purposes of registration.

Using FSL (Smith et al., 2004), we performed bias-field correction on all images to adjust for artifacts caused by the EEG wires. We performed slice-timing correction, motion correction, 0.01-Hz high-pass filtering, and 5-mm full width half max (FWHM) spatial smoothing on the functional data. The structural images were later used to align the functional data to a standard MNI brain.

### fMRI Data Processing for MVPA

Classifying brain-state on a trial-to-trial basis requires associating brain data to each trial. In slow block designs this can be done, for example, by averaging TR's within each block. The oddball detection tasks, however, are rapid event-related designs with relatively short ITI's (2–3 s). The temporal dynamics of the hemodynamic response function (HRF) evolve over much longer time-scales than the ITI, which results in significant overlap in BOLD response between adjacent trials. To un-mix these overlapping responses, we employed the LS-S deconvolution method proposed in [24]. For every trial, the time-series of each voxel is regressed against a “signal” regressor and a “noise” regressor. The “signal” regressor is the modeled HRF response due to that trial (a delta function centered at stimulus onset convolved with a canonical HRF), while the “noise” regressor is the modeled HRF response due to all other trials (superimposed linearly). The resulting regression coefficients of the “signal” regressor represent the estimated voxel activations due to that trial. It is important to note that only the trial timing information was used in this step, and not the label information, so there is no need to wrap this preprocessing step into the cross-validation procedure described in the “Model Selection” section below.

Although the exact number of voxels and trials varied for each subject, mean values were *n* = 368±1.2 (s.e.) trials and *p* = 51,804±859 (s.e.) voxels. The number of trials varied for each subject because trials missing a button press response were discarded, and trials for which the corresponding EEG data were corrupted were also discarded. Note that classification was performed for each subject in his/her ambient EPI image space (3×3×4 mm). When subsequently comparing across subjects, brain maps were transformed to the standard MNI brain space using the registrations derived from the structural scan.

Since the task involved a button press only for oddball trials, we expected classifiers derived from the full-brain to be dominated by motor areas. To instead identify regions that are specifically involved in the cognitive task, we additionally performed the classification on the brain data after excluding the “button press” network, which included postcentral and precentral gyrus, thalamus, cerebellum, caudate, putamen, and pallidum. These regions were identified using the MNI152 template brain. Secondary somatosensory cortex was preserved to avoid excluding the neighboring Heschl's gyrus, which was hypothesized to be important for the auditory oddball task. Overall, this reduced the number of features for this secondary classification analysis to *p* = 36,806±653 (s.e.) voxels. To differentiate between the two datasets, we refer to the full analysis as “whole brain”, and the secondary analysis as “without motor network.”

### Classification Method

Our analysis focuses on the classification problem of predicting the stimulus category (oddball/standard) from the full-brain fMRI data acquired during the experiment. We based our classification model on logistic regression, and treated each voxel as a feature. Thus, our goal is to learn a p-dimensional weight map w on the voxel space that defines a task discriminating “super-voxel.” To avoid overfitting and promote sparse models, we regularized our model by the elastic net penalty [19] so that our objective function may be expressed as:
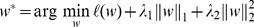
(1)where 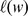 is the negative log-likelihood of the logistic regression model. Specifically, given a set of p-dimensional voxel activation maps 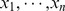 for a set of n trials and their associated labels 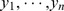 (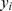 = 0 for standards and  = 1 for oddballs), 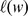 may be expressed as:
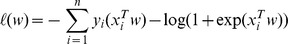



Although recent work in function-based registration methods has shown progress in aligning functional areas across subjects [25]–[28] classifiers were derived independently for each of the 14 subjects to avoid problems of inter-subject variability. In the Results section, we evaluate the inter-subject overlap of areas selected by the classifiers.

### Model Selection

For both the auditory and visual oddball tasks, classifiers were trained by 10-fold cross-validation, which was repeated on 10 random partitions. Classifier prediction accuracy was measured by the area under the ROC curve (A_z_), averaged over the 10 cross-validation runs.

Typically, model selection involves selecting the classifier with maximal cross-validated prediction accuracy. However, since interpretability of the brain map patterns is also important, we take a similar approach to [29] and consider a balance of prediction accuracy and reproducibility. Although there are many ways to define reproducibility, we focus on a measure of how robustly and reliably the sparse classifier selects voxels. Specifically, let 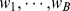 be a set of p-dimensional brain maps derived by training a classifier on B different training datasets. In this instance, each brain map corresponds to a result trained on one of the cross-validation folds, and B = 100. In general, however, the training sets may also be generated by bootstrap resampling [30]. From the B brain maps, we compute voxel selection probabilities 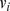 for each voxel 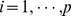 as the proportion of times that voxel was included in the model by the classifier. Ideally, 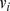 is either 0 or 1 for all voxels, corresponding to perfect voxel selection reliability. As a summary statistic for the classifier, we define the mean selection probability, 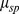 as:
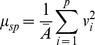
where 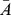 is the mean number of voxels selected by the classifier. This statistic may be interpreted as the expected selection probability of a voxel with nonzero weight selected at random from one of the brain maps. Note that since 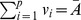, we have that 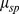 is bounded between 0 and 1, with 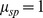 only for perfect voxel selection reliability (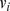 = 0 or 1 for all voxels).

Model selection is not as straightforward when considering both prediction accuracy (A_z_) and reproducibility (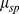). Unless the two objectives are perfectly correlated, choosing the best classifier entails a tradeoff, which, aside from being dependent on the application, may also be difficult to quantify. In this setting of multiple, possibly competing objectives, selecting a single “best” classifier is not well-defined; however, the set of candidate “best” classifiers are those that are Pareto optimal. These are classifiers that are not dominated by any other classifier, meaning that no other classifier achieves better performance on *all* of the objectives (reproducibility and prediction accuracy, in this case). For further reference on multi-objective optimization and Pareto optimality, see [31].

Three obvious Pareto optimal points to consider are: (1) the classifier with maximum reproducibility (MaxReprod), (2) the classifier with maximum prediction accuracy (MaxAz), and (3) the classifier that is closest to the optimal point of (reproducibility,prediction accuracy) = (1,1) (Joint sp)). In this paper, we focus on the latter two classifiers because it allows us to contrast the standard model selection method with a method that takes into account both objectives.

The reproducibility metric 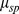 measures how robustly voxels are selected in aggregate, but it does not consider the variability in the weights assigned to these voxels. To take both sources of variability into account, we also consider the mean absolute z-score, 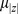. Given the 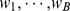 brain maps and corresponding voxel selection probabilities 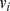, 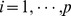, let 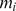 and 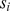 denote the mean and standard error of the weight for voxel i. A standard score can then be assigned by taking the ratio 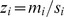. We then define 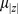 as:
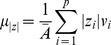
Thus, 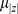 is a weighted average of the magnitude of voxel z-scores assigned by the classifier. Analogous to 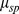 above, 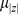 may be interpreted as the expected z-score magnitude of a voxel with nonzero weight selected at random from one of the brain maps. Note that, depending on how the B training sets were derived (e.g., by bootstrap or jackknife sampling), the bootstrap or jackknife estimate of variance can be used to estimate the standard errors 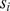 [30]. Since 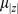 is not bounded to a specific range, it is not clear how to scale it in order to produce an appropriate trade-off against the prediction accuracy measure for model selection purposes. Moreover, there is no defined optimal (reproducibility,prediction accuracy) point. For these reasons, we only use 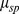 reproducibility measure when performing joint model selection. However, we show in the results section that, in aggregate, 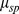 and 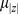 are very highly correlated, so that 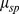 acts as a good surrogate measure.

## Results and Discussion

### Model Selection: Reproducibility vs. Prediction Accuracy Tradeoff

Classifiers were trained across a set of 1,100 paired values for 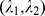 using 10-fold cross-validation repeated over 10 random partitions. Based on previous studies [13] and for memory considerations, we capped the maximum number of voxels to be included by a classifier at 1,000. This resulted in, for each pairing of regularization parameters, 100 brain maps from which to compute reproducibility maps (based on both voxel selection probability and z-scores), and cross-validated prediction accuracy was averaged across the 10 cross-validation runs. Unless stated otherwise, results on the auditory oddball experiment are presented in the main text, while those from the visual oddball experiment are provided in the supplementary material (see **Figure S1, Figure S2, Figure S3, Figure S4** and **Table S1, Table S2, Table S3**).

To assess statistical significance, we contrasted the results against a permutation test. For each subject, 300 permutations were generated by randomly permuting the response (stimulus category) across trials. The classifier was then re-trained for each permutation along the same set of regularization parameters and cross-validation partitions as the non-permuted case above. For the purposes of generating distributions of summary statistics of prediction accuracy and reproducibility under the null hypothesis of independence between data and response, permutations were pooled across subjects. Thus, to compute the distribution for prediction accuracy, for example, we recorded its maximum value attained over the grid of 1,100 regularization values for each of the 4,200 total permutations. From this distribution, one-tailed significance thresholds were computed (A_z_ = 0.60, p<0.05; A_z_ = 0.64, p<0.01). This process was repeated for the two reproducibility measures: (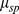 = 0.59, p<0.05; 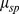 = 0.60, p<0.01) and (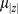 = 0.37, p<0.05; 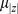 = 0.39, p<0.01).

**Figure 1:**
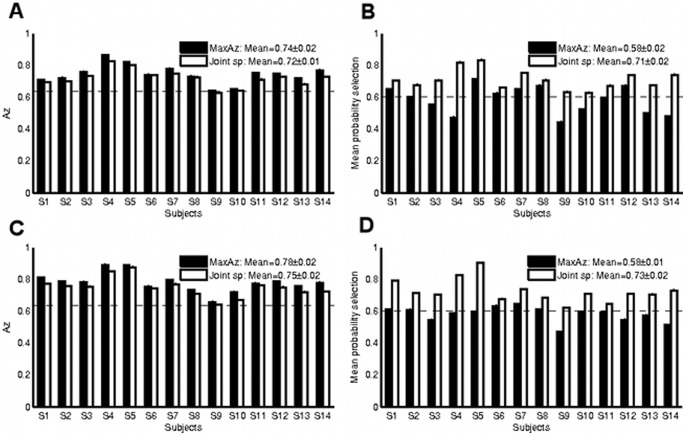
Comparison of summary statistic results for the MaxAz and Joint sp model selection methods on the auditory oddball data. Dotted horizontal lines indicate the p<0.01 significance levels. Cross-validated prediction accuracy (Az) results for each of 14 subjects under each of the model selection strategies are provided in **A** for the without motor network data and **C** for the whole brain data. For both model selection methods, prediction accuracy is significant at p<0.01 for all subjects. Reproducibility measure (mean probability of selection 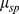) results for each of 14 subjects under each of the model selection strategies is provided in **B** for the without motor network data and **D** for the whole brain data. Here, a more drastic difference is noticeable between the two model selection strategies. While the Joint sp method is always above the p<0.01 line, the MaxAz method is significant at p<0.01 for only 7 (without motor network data) and 8 (whole brain data) of the 14 subjects.

**Figure 2:**
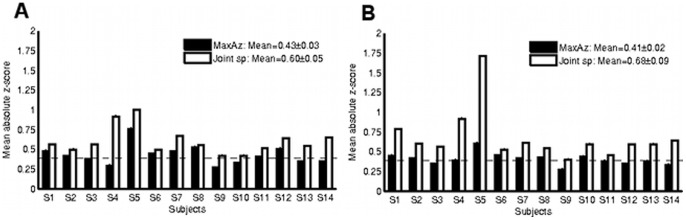
Comparison of reproducibility (mean absolute z-score 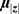) for both model selection methods on the auditory oddball without motor network data A and auditory oddball whole brain data B. Dotted horizontal lines indicate the p<0.01 significance levels. In both cases, reproducibility increases dramatically under Joint sp for many subjects.

**Figure 1** contrasts the summary statistics produced by the MaxAz and Joint sp model selection methods for each subject on the auditory oddball data. In each of the plots, the dotted horizontal lines indicate the p<0.01 significance thresholds. By definition, the MaxAz classifier will outperform the Joint sp method in terms of prediction accuracy (see **Figure 1A** and **Figure 1C**), but under-perform on the reproducibility (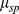) metric (see **Figure 1B** and **Figure 1D**). The more interesting characteristic of these plots is the degree of difference between the two methods –switching to the joint method incurs a relatively small loss in prediction accuracy in return for a much larger gain in reproducibility. Specifically, prediction accuracy does not fall under the p<0.01 significance line for any of the subjects in either method; in contrast, the joint method is always above p<0.01 in terms of reproducibility (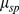), while the MaxAz method is above p<0.01 for only 8 (without motor network) and 7 (whole brain) of the 14 subjects. Moreover, mean (over subjects) prediction accuracy is within the margin of standard error between the two methods, while mean 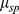 is significantly greater under the joint method. Interestingly, the joint method also improves reproducibility in terms of 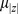 quite substantially (see **Figure 2A** and **Figure 2B**).

**Figure 3:**
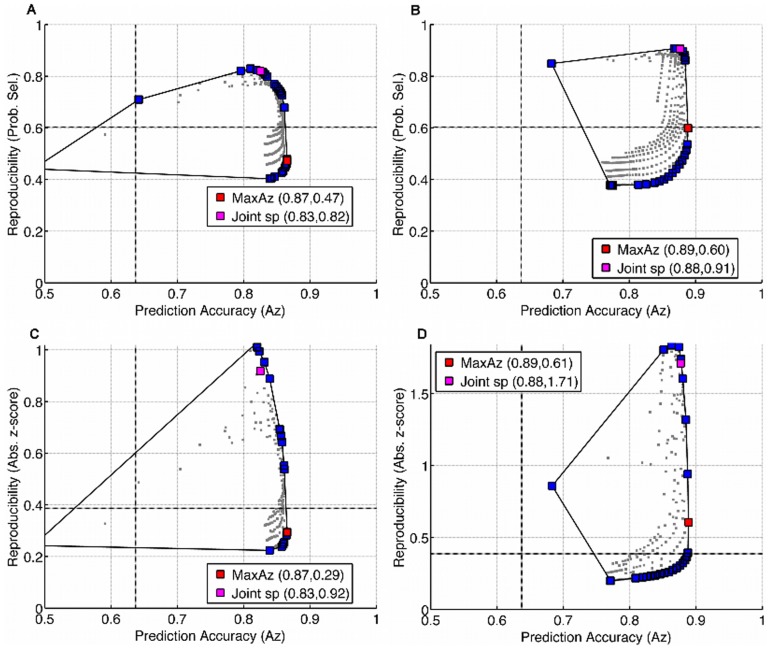
Reproducibility (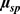) vs prediction accuracy (Az) curves for two subjects: A Subject S4 (without motor network), and B Subject S5 (whole brain). Reproducibility (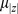) vs prediction accuracy curves for two subjects: C Subject S4 (without motor network), and D Subject S5 (whole brain). Thick lines indicate the p<0.01 significance thresholds. In each of the figures, the black curve delineates the convex hull of the 1,100 classifiers. Those classifiers that fall in the interior are plotted in gray, while those that lie on the boundary are highlighted. Despite the general trend of a positive correlation between reproducibility and prediction accuracy measures, the MaxAz (red) and Joint sp (magenta) model selection strategies select very different classifiers. In particular, the Joint sp method appears to tradeoff a small reduction in prediction accuracy for a much larger improvement in reproducibility.

To better visualize this tradeoff, **Figure 3** plots reproducibility (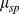) vs. prediction accuracy (A_z_) curves for two subjects. Since the most interesting classifiers lie on the boundary, the figures outline the convex hull of the 1,100 classifiers for each subject as a black curve, and those that lie on this boundary are highlighted. Other classifiers that lie on the interior of the convex hull are plotted in gray. The classifiers corresponding to the MaxAz and Joint sp methods are highlighted in red and magenta, respectively. An interesting characteristic of these plots is that although there is a positive correlation trend between the two objectives, the model selection methods tend to select very different classifiers. Specifically, their prediction accuracies are similar but reproducibility scores are much more variable. This suggests that there are a number of models to choose from with competitive prediction accuracies, but widely varying reproducibility scores. Thus, selecting based on prediction accuracy alone is susceptible to marginally improving prediction accuracy at the expense of drastically reducing reproducibility. The joint method appears to overcome this limitation and improve robustness. This is particularly true for subjects S4 and S5 (see **Figure 3A** and **Figure 3B**), in which the improvement in reproducibility is most dramatic.

We also assessed the relationship between the two reproducibility measures, 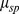 and 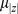. As outlined in Section 4.3, only 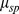 was considered in the model selection stage even though 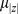 is a more informative measure of reproducibility. It turns out that both measures are highly correlated, so that one may be used as a surrogate for the other. Across all subjects and datasets, the correlation between the two measures was never less than 0.95. **Figure 3C** and **Figure 3D** verify that the Joint sp method selects nearly optimal classifiers when reproducibility is evaluated as 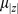.

### Voxel-Based Significance Analysis

The reproducibility measures considered so far provide summaries over the entire brain, but lack specificity on which clusters of voxels in particular are selected by the classifier. In this section, we assess reliability in terms of the anatomical coordinates of selected voxels. We obtained distributions for voxel-specific selection probabilities and z-scores under the null hypothesis by returning to the permutation analysis described in the previous section. Since there are no spatial priors and voxels are treated equally by the classifier, we assumed that the voxel statistics were identically distributed, which allowed us to pool across voxels. For each permutation, its best classifier was selected using the Joint sp method, and the corresponding z-scores and selection probabilities of any voxels with selection probability v_i_>0 were used to build the distributions.

To evaluate voxel-specific significance of a brain map, we first converted the voxel statistics (whether they be z-scores or selection probabilities) of any voxel with v_i_>0 to p-values based on the null distributions generated above. We then thresholded the brain map at false discovery rate (FDR) *α* = 0.05 [32]. Specifically, let 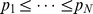 denote the ordered p-values of the N voxels with nonzero selection probabilities. Then voxels 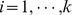 are deemed significant, where k satisfies:
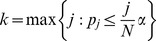
This controls the expected rate of false discoveries at *α*. This FDR analysis was computed with respect to both voxel selection probabilities and voxel z-scores.

**Table 1** lists the number of significant voxels selected by the selection probability and z-score FDR analysis for both the auditory oddball without motor network and auditory oddball whole brain data. Significant voxels were found for both data except for subjects S9 and S10. Interestingly, these subjects also had the weakest prediction accuracies (see **Figure 1A** and **Figure 1C**). The selection probability analysis always selects more voxels as significant, and we verified empirically that the voxels selected by the z-score analysis were always a subset of those selected by the selection probability analysis. This suggests a hierarchy of significance testing, in which the selection probabilities may be used to identify broad regions that contribute consistently, while the z-scores further refine this to the most reliable and focal regions. In this light, we view both analyses as informative.

**Table 1:** Number of significant voxels selected for each of 14 subjects.

Auditory oddball without motor network							
	# sig (|z|)	# sig (sp)	A		# sig (|z|)	# sig (sp)	A
S1	44	123	633	S8	3	18	91
S2	23	148	993	S9	0	82	877
S3	51	156	808	S10	0	0	990
S4	125	141	312	S11	29	134	870
S5	46	49	104	S12	71	141	627
S6	12	76	461	S13	18	45	277
S7	117	190	720	S14	24	56	234

Auditory oddball whole brain							
	# sig (|z|)	# sig (sp)	A		# sig (|z|)	# sig (sp)	A
S1	25	25	68	S8	21	94	521
S2	74	214	914	S9	2	72	853
S3	72	168	890	S10	7	17	82
S4	317	347	755	S11	18	119	934
S5	59	73	99	S12	61	161	780
S6	24	138	775	S13	19	44	223
S7	90	230	963	S14	43	109	433

# sig (|z|) and # sig (sp) denote the number of voxels deemed significant at FDR = 0.05 when testing z-scores and selection probabilities, respectively. “A” denotes the average number of voxels selected.

**Figure 4:**
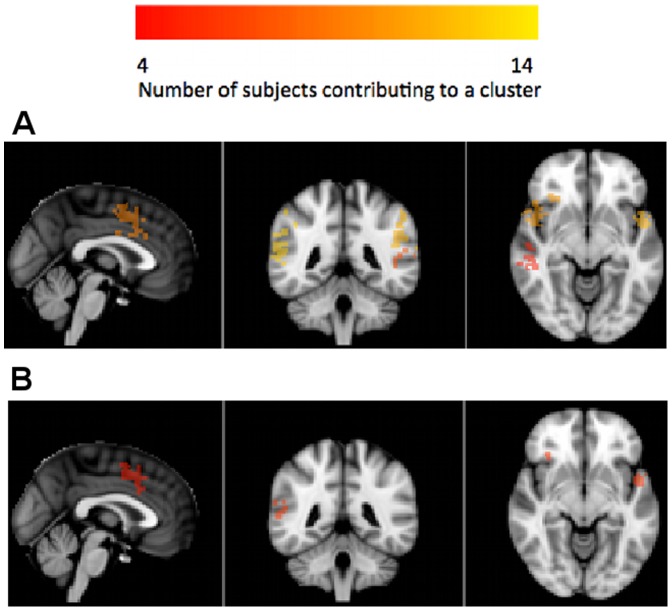
Group-level brain reproducibility maps evaluated on the auditory oddball without motor network data (MNI coordinates: (0,−44,−8), R-L orientation). Reproducibility was evaluated at the voxel level by testing each voxel's probability of selection or absolute z-score statistic against a null distribution generated by a permutation test. Subject-specific significance masks were created by thresholding at FDR α = 0.05. After transforming to MNI space, masks were summed so that the value at each voxel equals the number of subjects that declare it to be significant. This group mask was then spatially clustered and each cluster reports the total number of subjects that contributed to it. (a) Clusters from the selection probability statistic on the without motor network data; (b) Clusters from the absolute z-score statistic on the without motor network data. Associated regions are listed in **Table 2**.

**Figure 5:**
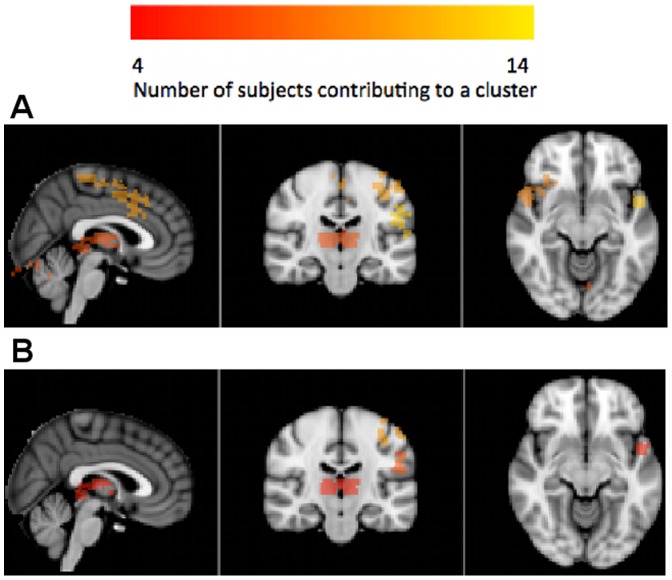
Group-level brain reproducibility maps evaluated on the auditory oddball whole brain data (MNI coordinates: (2,−20,−10), R-L orientation). Reproducibility was evaluated at the voxel level by testing each voxel's probability of selection or absolute z-score statistic against a null distribution generated by a permutation test. Subject-specific significance masks were created by thresholding at FDR α = 0.05. After transforming to MNI space, masks were summed so that the value at each voxel equals the number of subjects that declare it to be significant. This group mask was then spatially clustered and each cluster reports the total number of subjects that contributed to it. (a) Clusters from the selection probability statistic on the whole brain data; (b) Clusters from the absolute z-score statistic on the whole brain data. The absolute z-score method appears to select a more focal subset. Associated regions are listed in **Table 3**.

To evaluate the regional location and inter-subject spatial overlap, we first transformed the FDR-thresholded brain maps of each subject into MNI space and generated subject-specific brain masks of significant voxels. These masks were then summed over subjects so that the value at each voxel equals the number of subjects that declare it to be significant. Since we do not expect inter-subject spatial overlap on a voxel-by-voxel basis, we instead clustered this group mask and reported the number of subjects that contribute to each of the clusters. The cluster atlas labels, sizes, total number of subjects contributing per cluster, and the maximum number of subjects contributing to a given voxel in the cluster are listed in **Table 2** and **Table 3**. Note that since the MNI space is at a higher spatial resolution, the sizes of clusters are inflated. Brain map figures are also provided in **Figure 4** and **Figure 5**.

**Table 2:** Group-level clusters of significant voxels on the auditory oddball without motor network data.

Auditory oddball without motor network			
Using voxel-level probability of selection statistic			
Region	Size	Total # Subjects	Max Subj/Voxel
Central Opercular Cortex (L)	252	6	3
Insular Cortex (R)	162	6	2
Angular Gyrus (R)	133	6	2
Cingulate Gyrus (A)	445	5	2
Parietal Opercular Cortex (L)	145	5	2
Temporal Pole (R)	129	4	2

Using voxel-level absolute z-score statistic			
Region	Size	Total # Subjects	Max Subj/Voxel
Angular Gyrus (R)	797	12	3
Central Opercular Cortex (L)	1156	11	3
Insular Cortex (R)	640	10	3
Cingulate Gyrus (A)	745	9	3
Middle Temporal Gyrus (L)	354	7	2
Middle Temporal Gyrus (R)	352	5	2
Precuneous Cortex (R)	167	5	3
Superior Frontal Gyrus (L)	241	4	3
Cingulate Gyrus (P)	102	4	2

Associated brain map figures are provided in **Figure 4** and **Figure 5**. Notation: (L) – left-lateralized, (R) right-lateralized, (A) anterior, (P) posterior.

**Table 3:** Group-level clusters of significant voxels on the auditory oddball whole brain data.

Auditory oddball whole brain			
Using voxel-level probability of selection statistic			
Region	Size	Total # Subjects	Max Subj/Voxel
Central Opercular Cortex (L)	1153	12	3
Postcentral Gyrus (L)	1020	10	3
Cingulate Gyrus (A)	935	10	3
Insular Cortex (R)	695	9	3
Angular Gyrus (R)	410	9	3
Thalamus (R)	1210	7	4
Cerebellum (R)	262	7	2
Middle Temporal Gyrus (L)	122	7	2
Postcentral Gyrus (R)	528	4	2
Cerebellum (R)	311	4	2

Using voxel-level absolute z-score statistic			
Region	Size	Total # Subjects	Max Subj/Voxel
Postcentral Gyrus (L)	637	10	2
Postcentral Gyrus (L)	271	7	2
Supramarginal Gyrus (R)	157	6	2
Thalamus (L)	981	5	2
Central Opercular Cortex (L)	120	5	2
Cingulate Gyrus (A)	403	4	2
Temporal Pole (R)	144	4	2

Associated brain map figures are provided in **Figure 4** and **Figure 5**. Notation: (L) – left-lateralized, (R) – right-lateralized, (A) anterior, (P) posterior.

As expected for the whole brain data, we found large clusters in regions related to the button press, including thalamus, cerebellum, and left (contralateral) postcentral gyrus. For both datasets, discriminating activity was found in central opercular cortex, extending to include auditory regions. Insular cortices, anterior cingulate, and angular gyrus were also consistently selected in both datasets; these areas are commonly associated with the P300 EEG response that is reliably generated in such oddball decision-making tasks [33]. By excluding the motor network, we detected additional discriminative regions that have been linked to auditory target detection in fMRI data, including the posterior cingulate and right middle temporal gyrus [34].

### Summary/Conclusion

We have described an approach for leveraging permutation testing and bootstrapping, together with a method for fast simultaneous training of generalized linear models (FaSTGLZ) to construct a large family of classifiers that we subsequently mapped into a utility space. Within this space optimal classifiers can be identified by considering their joint decoding accuracy and reproducibility. As multivariate decoding models become more prevalent in neuroimaging, and as the dimensions these datasets increase, it is ever more important to systematically explore the accuracy/reproducibility tradeoff. Finally, our methods extend to a wide range of applications of decoding models, from basic exploratory data analysis and inference in cognitive neuroscience to brain computer interfaces and neurofeedback systems.

## Supporting Information

Figure S1
**Comparison of summary statistic results for the MaxAz and Joint sp model selection methods on the visual oddball without motor network data.** Dotted horizontal lines indicates the p<0.01 significance thresholds. Cross-validated prediction accuracy (Az) results for each of 14 subjects under each of the model selection strategies. For both model selection strategies are provided in (a) for the without motor network data and (c) for the whole brain data. For both model selection methods, prediction accuracy is significant at p<0.01 for all subjects. Reproducibility measure (mean probability of selection 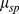) results for each of 14 subjects under each of the model selection strategies is provided in (b) for the without motor network data and (d) for the whole brain data. Here, a more drastic difference is noticeable between the two model selection strategies. While the Joint sp method is always above the p<0.01 line, the MaxAz method is significant at p<0.01 for only 4 (without motor network data) and 5 (whole brain data) of the 14 subjects.(TIF)

Figure S2
**Comparison of reproducibility (mean absolute z-score** 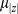 **) for both model selection methods on the visual oddball without motor network data (a) and visual oddball whole brain data (b).** Dotted horizontal lines indicates the p<0.01 significance thresholds. In both cases, reproducibility increases dramatically under Joint sp for many subjects.(TIF)

Figure S3 **Group-level brain reproducibility maps evaluated on the visual oddball without motor network data (MNI coordinates: (0,18,8), R-L orientation).** For each of 14 subjects, reproducibility was evaluated at the voxel level by testing each voxel's probability of selection or absolute z-score statistic against a null distribution generated by a permutation test. Subject-specific significance masks were then created by thresholding at false discovery rate α = 0.05 to correct for multiple comparisons. After transforming to MNI space, masks were summed so that the value at each voxel equals the number of subjects that declare it to be significant. This group mask was then spatially clustered and each cluster reports the total number of subjects that contributed to it. (a) Group-level clusters derived using the selection prob-ability statistic; (b) Group-level clusters derived using the absolute z-score statistic. The absolute z-score method appears to select a more focal subset. Associated regions are listed in **Table S2**.
(TIF)

Figure S4 **Group-level brain reproducibility maps evaluated on the visual oddball whole brain data (MNI coordinates: (0,−18,18), R-L orientation).** For each of 14 subjects, reproducibility was evaluated at the voxel level by testing each voxel's probability of selection or absolute z-score statistic against a null distribution generated by a permutation test. Subject-specific significance masks were then created by thresholding at false discovery rate α = 0.05 to correct for multiple comparisons. After transforming to MNI space, masks were summed so that the value at each voxel equals the number of subjects that declare it to be significant. This group mask was then spatially clustered and each cluster reports the total number of subjects that contributed to it. (a) Group-level clusters derived using the selection probability statistic; (b) Group-level clusters derived using the absolute z-score statistic. The absolute z-score method appears to select a more focal subset. Associated regions are listed in Table S3.
(TIF)

Table S1 **Number of significant voxels selected for each of 14 subjects.** # sig (|z|) and # sig (sp) denote the number of voxels deemed significant at FDR = 0.05 when testing z-scores and selection probabilities, respectively. “A” denotes the average number of voxels selected.
(DOC)

Table S2 **Group-level clusters of significant voxels on the auditory oddball without motor network data.** Associated brain map figures are provided in **Figure S3**. Notation: (L) – left-lateralized, (R) right-lateralized, (A) anterior, (P) posterior.
(DOC)

Table S3 **Group-level clusters of significant voxels on the auditory oddball whole brain data.** Associated brain map figures are provided in **Figure S4**. Notation: (L) – left-lateralized, (R) right-lateralized, (A) anterior, (P) posterior.
(DOC)
