# Supplementary figures and images for: Fast Bootstrapping and Permutation Testing for Assessing Reproducibility and Interpretability of Multivariate fMRI Decoding Models
Source: PLoS One. 2013 Nov 14;8(11):e79271. doi: 10.1371/journal.pone.0079271 (PMC3828388; doi:10.1371/journal.pone.0079271)

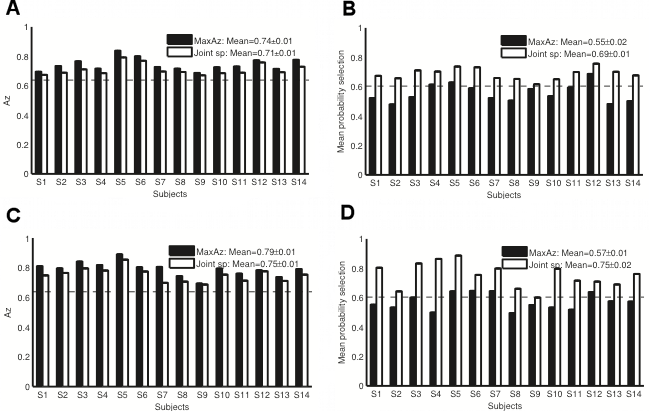

Supplement: Figure S1 — Comparison of summary statistic results for the MaxAz and Joint sp model selection methods on the visual oddball without motor network data. Dotted horizontal lines indicates the p<0.01 significance thresholds. Cross-validated prediction accuracy (Az) results for each of 14 subjects under each of the model selection strategies. For both model selection strategies are provided in (a) for the without motor network data and (c) for the whole brain data. For both model selection methods, prediction accuracy is significant at p<0.01 for all subjects. Reproducibility measure (mean probability of selection ) results for each of 14 subjects under each of the model selection strategies is provided in (b) for the without motor network data and (d) for the whole brain data. Here, a more drastic difference is noticeable between the two model selection strategies. While the Joint sp method is always above the p<0.01 line, the MaxAz method is significant at p<0.01 for only 4 (without motor network data) and 5 (whole brain data) of the 14 subjects. (TIF) [file pone.0079271.s001.tif]

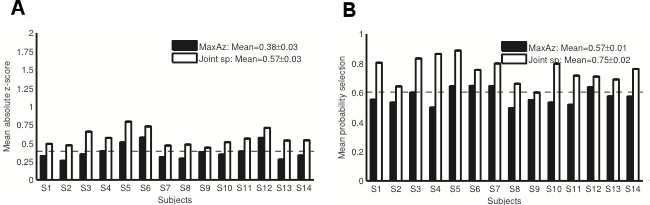

Supplement: Figure S2 — Comparison of reproducibility (mean absolute z-score ) for both model selection methods on the visual oddball without motor network data (a) and visual oddball whole brain data (b). Dotted horizontal lines indicates the p<0.01 significance thresholds. In both cases, reproducibility increases dramatically under Joint sp for many subjects. (TIF) [file pone.0079271.s002.tif]

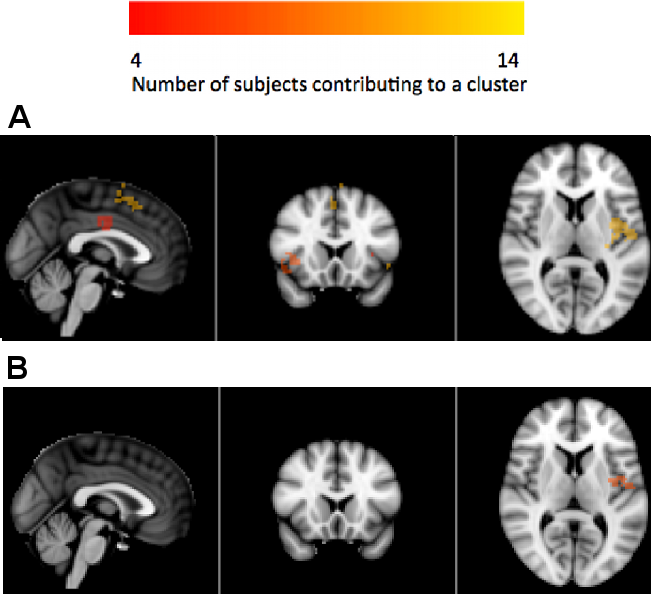

Supplement: Figure S3 — Group-level brain reproducibility maps evaluated on the visual oddball without motor network data (MNI coordinates: (0,18,8), R-L orientation). For each of 14 subjects, reproducibility was evaluated at the voxel level by testing each voxel's probability of selection or absolute z-score statistic against a null distribution generated by a permutation test. Subject-specific significance masks were then created by thresholding at false discovery rate α = 0.05 to correct for multiple comparisons. After transforming to MNI space, masks were summed so that the value at each voxel equals the number of subjects that declare it to be significant. This group mask was then spatially clustered and each cluster reports the total number of subjects that contributed to it. (a) Group-level clusters derived using the selection prob-ability statistic; (b) Group-level clusters derived using the absolute z-score statistic. The absolute z-score method appears to select a more focal subset. Associated regions are listed in Table S2. (TIF) [file pone.0079271.s003.tif]

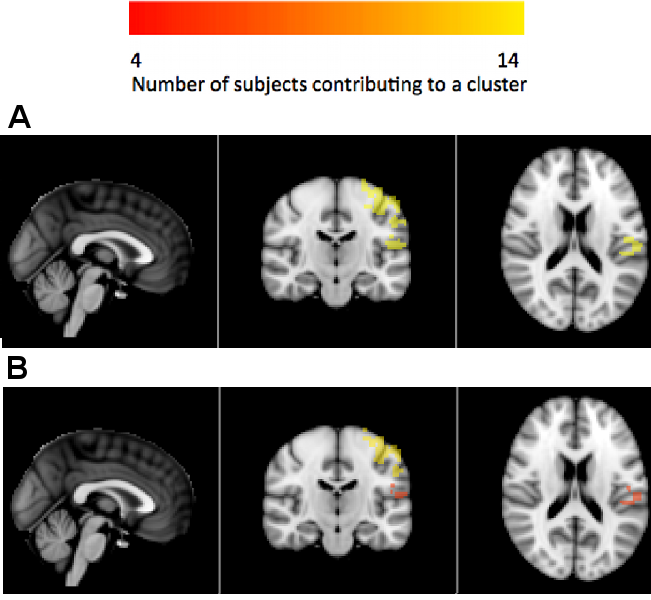

Supplement: Figure S4 — Group-level brain reproducibility maps evaluated on the visual oddball whole brain data (MNI coordinates: (0,−18,18), R-L orientation). For each of 14 subjects, reproducibility was evaluated at the voxel level by testing each voxel's probability of selection or absolute z-score statistic against a null distribution generated by a permutation test. Subject-specific significance masks were then created by thresholding at false discovery rate α = 0.05 to correct for multiple comparisons. After transforming to MNI space, masks were summed so that the value at each voxel equals the number of subjects that declare it to be significant. This group mask was then spatially clustered and each cluster reports the total number of subjects that contributed to it. (a) Group-level clusters derived using the selection probability statistic; (b) Group-level clusters derived using the absolute z-score statistic. The absolute z-score method appears to select a more focal subset. Associated regions are listed in Table S3. (TIF) [file pone.0079271.s004.tif]
