# Supplementary material for: Fast Bootstrapping and Permutation Testing for Assessing Reproducibility and Interpretability of Multivariate fMRI Decoding Models
Source: PLoS One. 2013 Nov 14;8(11):e79271. doi: 10.1371/journal.pone.0079271 (PMC3828388; doi:10.1371/journal.pone.0079271)
Supplement: Table S1 — Number of significant voxels selected for each of 14 subjects. # sig (|z|) and # sig (sp) denote the number of voxels deemed significant at FDR = 0.05 when testing z-scores and selection probabilities, respectively. “A” denotes the average number of voxels selected. (DOC) [file pone.0079271.s005.doc]

**Reproducibility of Decoding Models in fMRI Multivariate Pattern Analyses**

**Supplementary Material**

Bryan R. Conroy, Jennifer M. Walz, Paul Sajda

Table S1: Number of significant voxels selected for each of 14 subjects. # sig (|z|) and # sig (sp) denote the number of voxels deemed significant at FDR=0.05 when testing z-scores and selection probabilities, respectively. “A” denotes the average number of voxels selected.

| Visual oddball without motor network | | | | | | | | |
| --- | --- | --- | --- | --- | --- | --- | --- | --- |
|  | # sig (|z|) | # sig (sp) | A |  |  | # sig (|z|) | # sig (sp) | A |
| S1 | 22 | 139 | 972 |  | S8 | 20 | 129 | 862 |
| S2 | 6 | 108 | 861 |  | S9 | 5 | 128 | 995 |
| S3 | 8 | 17 | 55 |  | S10 | 7 | 17 | 126 |
| S4 | 30 | 114 | 518 |  | S11 | 18 | 77 | 384 |
| S5 | 13 | 15 | 53 |  | S12 | 11 | 13 | 54 |
| S6 | 14 | 20 | 76 |  | S13 | 3 | 38 | 221 |
| S7 | 13 | 121 | 999 |  | S14 | 46 | 168 | 996 |
| Visual oddball whole brain | | | | | | | | |
|  | # sig (|z|) | # sig (sp) | A |  |  | # sig (|z|) | # sig (sp) | A |
| S1 | 14 | 20 | 52 |  | S8 | 14 | 110 | 739 |
| S2 | 20 | 116 | 948 |  | S9 | 4 | 51 | 582 |
| S3 | 26 | 26 | 51 |  | S10 | 41 | 52 | 178 |
| S4 | 34 | 40 | 69 |  | S11 | 52 | 86 | 424 |
| S5 | 100 | 105 | 164 |  | S12 | 4 | 15 | 57 |
| S6 | 47 | 66 | 252 |  | S13 | 4 | 48 | 269 |
| S7 | 38 | 38 | 122 |  | S14 | 15 | 27 | 94 |
