# Supplementary material for: Fast Bootstrapping and Permutation Testing for Assessing Reproducibility and Interpretability of Multivariate fMRI Decoding Models
Source: PLoS One. 2013 Nov 14;8(11):e79271. doi: 10.1371/journal.pone.0079271 (PMC3828388; doi:10.1371/journal.pone.0079271)
Supplement: Table S2 — Group-level clusters of significant voxels on the auditory oddball without motor network data. Associated brain map figures are provided in Figure S3. Notation: (L) – left-lateralized, (R) right-lateralized, (A) anterior, (P) posterior. (DOC) [file pone.0079271.s006.doc]

**Reproducibility of Decoding Models in fMRI Multivariate Pattern Analyses**

**Supplementary Material**

Bryan R. Conroy, Jennifer M. Walz, Paul Sajda

Table S2: Group-level clusters of significant voxels on the auditory oddball without motor network data. Associated brain map figures are provided in Figure S3. Notation: (L) – left-lateralized, (R) right-lateralized, (A) anterior, (P) posterior.

| Visual oddball without motor network | | | |
| --- | --- | --- | --- |
| Using voxel-level probability of selection statistic | | | |
| Region | Size | Total # Subjects | Max Subj/Voxel |
| Central Opercular Cortex (L) | 982 | 11 | 3 |
| Juxtapositional Lobule Cortex (L) | 456 | 11 | 3 |
| Insular Cortex (R) | 158 | 7 | 3 |
| Angular Gyrus (L) | 160 | 6 | 2 |
| Supramarginal Gyrus (L) | 157 | 6 | 3 |
| Cingulate Gyrus (A) | 224 | 5 | 2 |
| Frontal Orbital Cortex (L) | 206 | 5 | 3 |
| Middle Temporal Gyrus (R) | 185 | 4 | 1 |
| Lateral Occipital Cortex (L) | 149 | 4 | 1 |
| Planum Temporale (R) | 100 | 4 | 2 |
| Using voxel-level absolute z-score statistic | | | |
| Region | Size | Total # Subjects | Max Subj/Voxel |
| Central Opercular Cortex (L) | 193 | 7 | 2 |
| Parietal Operculum Cortex (L) | 102 | 4 | 2 |
