# Supplementary material for: Fast Bootstrapping and Permutation Testing for Assessing Reproducibility and Interpretability of Multivariate fMRI Decoding Models
Source: PLoS One. 2013 Nov 14;8(11):e79271. doi: 10.1371/journal.pone.0079271 (PMC3828388; doi:10.1371/journal.pone.0079271)
Supplement: Table S3 — Group-level clusters of significant voxels on the auditory oddball whole brain data. Associated brain map figures are provided in Figure S4. Notation: (L) – left-lateralized, (R) right-lateralized, (A) anterior, (P) posterior. (DOC) [file pone.0079271.s007.doc]

**Reproducibility of Decoding Models in fMRI Multivariate Pattern Analyses**

**Supplementary Material**

Bryan R. Conroy, Jennifer M. Walz, Paul Sajda

Table S3: Group-level clusters of significant voxels on the auditory oddball whole brain data. Associated brain map figures are provided in Figure S4. Notation: (L) – left-lateralized, (R) right-lateralized, (A) anterior, (P) posterior.

| Visual oddball whole brain | | | |
| --- | --- | --- | --- |
| Using voxel-level probability of selection statistic | | | |
| Region | Size | Total # Subjects | Max Subj/Voxel |
| Postcentral Gyrus (L) | 2233 | 14 | 5 |
| Cerebellum (L) | 80 | 5 | 2 |
| Juxtapositional Lobule Cortex (L) | 142 | 4 | 2 |
| Using voxel-level absolute z-score statistic | | | |
| Region | Size | Total # Subjects | Max Subj/Voxel |
| Postcentral Gyrus (L) | 1399 | 13 | 4 |
| Central Opercular Cortex (L) | 238 | 6 | 2 |
